# Editorial

**Published:** 1839-02-23

**Authors:** 


### THE MEDICAL EXAMINER.

PHILADELPHIA, FEB. 23, 1839.

We observe by the last French journals, that Prof. Andral will succeed Broussais in the chair of General Pathology and Therapeutics. Broussais was never able to give the subject its full developement. His mind had been so long directed towards a particular class of facts, that it had acquired a bias, which unfitted him for a department in which the instructer must assume the widest range consistent with sound philosophy.

Dr. Andral unites many qualifications. He has great familiarity with the phenomena of disease, and an extensive acquaintance with medical literature in general, while his mind is of that high order, which can take hold of a subject and view it in all its relations, by rapidly seizing a few prominent characters. We rejoice that the chair of this department of science will be so ably supplied,—its influence will certainly be widely felt, extending to all the branches of medical instruction; but, in unskilful hands, nothing can be more utterly insignificant than the chair of General Pathology.

---

On Wednesday evening, the 20th inst., Dr. Randolph read before the Medical Society, the memoir of the late Dr. Physick, prepared in compliance with the request of the society. It was listened to with great pleasure by a very numerous and respectable body of physicians; and we have the satisfaction to say, that a copy has been furnished the society for publication. As might have been anticipated, Dr. Randolph has presented to the profession, a faithful and most interesting picture of the life and professional career of the Father of American Surgery. We promise ourselves the pleasure of offering our readers an extended notice of the memoir when published.

